# Training and Management Status of Wound, Ostomy, and Continence (WOC) Nurses: A Cross-Sectional Survey From China

**DOI:** 10.7759/cureus.23793

**Published:** 2022-04-03

**Authors:** Min Zhang, Di Wei, Xiaomei Zhu, Yongyi Chen

**Affiliations:** 1 Wound Care, Hunan Cancer Hospital, Changsha, CHN

**Keywords:** education, current situation, management, training, woc nurses

## Abstract

Objective: To investigate the current situation with regard to training and management of wound, ostomy, and continence (WOC) nurses in China.

Methods: A self-designed questionnaire was used to investigate the training and management of WOC nurses.

Results: A total of 218 WOC nurses received cross-sectional surveys. Out of these nurses, 57.30% graduated from the International Stoma Therapists School, 22.90% were full-time WOC nurses, only 24.80% had engaged in funded research, and 26.10% had an additional allowance. There is room for further improvement in the field of education, scientific research, and self-development.

Conclusion: WOC work has developed rapidly in China, and there have been some achievements in personnel training, specialized nursing, teaching, and scientific research.

## Introduction

Due to the aging population and the increasing incidence of chronic diseases, such as malignant tumors and diabetes, the number of patients with chronic wounds, various causes of ostomy, and potential incontinence is growing year on year, creating new requirements for specialized wound, ostomy, and continence (WOC) nurses globally [1]. The term “WOC nurses” refers to the specialized nursing staff engaged in the field of WOC nursing, preventing and treating complications, providing psychological and rehabilitation guidance, and other counseling services for patients and their families [2]. In developed countries such as the United States, the United Kingdom, and Japan, the training and management system of specialized WOC nurses is relatively mature, with standardized selection conditions, training materials, a qualification certification system, post setting, etc. [3,4]. In recent years, China has developed rapidly, and the number of specialized nurses is increasing [5]. However, there are so many people in need, and economic development and medical conditions are not balanced; the training and work situations of specialized WOC nurses are different, and the role played in the development of specialized nursing is also uneven [6]. To better understand the work and professional development of China’s specialized WOC nurses in their respective hospitals, to sum up and analyze the experience and difficulties in their practice, to promote the sustained and positive development of specialized WOC nurses’ training, and to maximize the leading role of high-level nursing talents in the development of specialized nursing, we investigated the specialized WOC nurses attending the 2019 National Wound Ostomy Continence Forum (WOCF) Conference.

## Materials and methods

Data collection

The study used the convenience sampling method to collect data from WOC specialist nurses who attended the 2019 National WOCF Conference. The inclusion criteria included nurses who have obtained a qualification certificate, registered nurses who have obtained enterostomal therapist (ET) certification, and nurses who have worked for one year or more.

Clinicians undertaking WOC care completed the self-designed paper questionnaire for specialist WOC nurses, though an online version of the form was also available. Paper forms were scanned and the data were extracted using survey software Sojump (Zhongdian, Shandong, China). All paper surveys were stored securely until scanning and then shredded immediately following transcription. Data were analyzed for the purposes of providing a more generalizable insight into the general condition and working situation of specialist WOC nurses. The working situation includes 22 items in total, comprising the position, work content, work scope, management mode, remuneration packages, etc. To ensure the nurses’ anonymity, no identifiers were used. It was therefore not possible to run retrospective data queries to determine missing data values.

Data analysis

After data collection and preprocessing, SPSS version 18.0 (SPSS Inc., Chicago, IL) was used to calculate the percentage, mean, and standard deviation to describe the data statistically.

## Results

A total of 227 questionnaires were sent out and 218 valid questionnaires were recovered. The effective recovery rate was 96.04%.

General information of specialist WOC nurses

Of the 218 specialist nurses involved in the survey, 216 were women (99.10%) and two were men (0.90%). The age range was 23-58 (36.45 ± 6.43) years and the range of years in the job was 3-41 (15.84 ± 7.69) years. With regard to education level, the majority (193, 90.2%) had undergraduate degrees; there were 12 graduate students (5.50%) and 13 junior college students (6.00%). With regard to professional positions held, 119 (54.60%) were supervisor nurses, 50 (22.90%) were co-chief superintendent nurses, and 49 (22.48%) were nurse practitioners. A total of 159 (72.90%) specialist nurses worked in tertiary hospitals and 59 (27.04%) in secondary hospitals. Of the nurses, 125 (57.30%) were trained by the International Stoma Therapists School, 15 (6.90%) by the Chinese Nursing Association, 42 (19.30%) by provincial nursing associations, and 36 (16.50%) by other training institutions.

Working situation of specialist WOC nurses

In this survey, there were 128 (58.70%) nurses who had no job, while 90 (41.28%) were managers. There were 50 (22.90%) specialized nurses working full-time, 126 (57.80%) working part-time, and 42 (19.30%) were not involved in WOC. The other items are shown in Table 1.

**Table 1:** Training and working status of specialist WOC nurses. WOC: wound, ostomy, and continence.

	N (%）
Availability of training to meet needs	
Very satisfying	84 (38.50)
Basically satisfying	102 (46.80)
Partially satisfying	22 (10.10)
Not satisfying	10 (4.60)
Source of work guidance	
Previous experience	52 (23.90)
Enterostomal therapist course training	114 (52.30)
Guidelines and books	35 (16.10)
Others	17 (7.80)
Scope of work	
Hospital-wide	106 (48.60)
Only in some hospital departments	40 (18.30)
Only in the ward	56 (25.70)
Inside and outside the hospital	16 (7.30)
Access to scientific research funds	
Yes	54 (24.80)
No	164 (75.20)
Hospital management model	
Direct management by the nursing department	91 (44.50)
Management by clinical departments	86 (39.40)
Outpatient management	23 (10.60)
Others	12 (5.50)
Is there a performance appraisal system?	
Yes	91 (41.70)
No	127 (58.30)
Whether there is a promotion system for professional titles	
Yes	79 (36.20)
No	139 (63.80)
Is there a remuneration system?	
Yes	77 (35.30)
No	141 (64.70)
Is there a retraining certification system?	
Yes	102 (46.80)
No	116 (53.20)
Is there a job responsibility system?	
Yes	113 (61.00)
No	85 (39.00)
Whether or not to open a WOC clinic	
Yes	127 (58.30）
No	91 (41.70）
Is there an additional allowance?	
Yes	57 (26.10)
No	181 (73.90)
Are there more opportunities for further education?	
Yes	178 (81.70)
No	40 (18.30)

## Discussion

Present situation of specialist WOC nurses

At present, specialized domestic WOC nurses are, on average, young and middle-aged, and their average working life is longer than that of other nurses, which indicates that they have rich clinical experience and good development prospects. Among the specialized nurses, 94.00% hold a bachelor's degree or above, and 77.50% are supervisor nurses or above, which indicates that the overall quality of the specialized nurses in the nursing team is high, and they are the backbone of their nursing teams. However, although at present specialist domestic WOC nurses are mainly undergraduates, this education level is still lower than the basic education requirements for specialist WOC nurses in the United States, which is a master’s degree [7]. The reason is that the overall education of Chinese nurses is on the lower side. In recent years, with the continuous improvement of the educational level of nurses in our country, we can strengthen the on-the-job education of specialized WOC nurses and gradually improve their educational level. The percentage of specialist WOC nurses who work in tertiary hospitals is 72.90%, while more than 40% of specialist nurses in the United States carry out practical activities in the community [8]. At present, it is difficult for community nurses to meet the selection criteria for specialist WOC nurses training in China, but they can train to become community nurses, or we can encourage specialist nurses to participate in community lectures, engage in free social consultations, and gradually broaden the scope of nursing services for specialist WOC nurses [9]. Because higher nursing education in China does not involve specialization in WOC, in-service nurses lack a standard model and concept of WOC nursing, so the International Stoma Therapists School plays an important role in the training of specialized WOC nurses [10]. In this survey, 57.30% of the specialist nurses graduated from the International Stoma Therapists School. As the first standardized training institution for specialized nurses in China, the International Stoma Therapists School has set a model for the standardized development of specialized nurses in China, and also provided a reference model for the development of other specialized nursing [11].

Present working situation of domestic specialist nurses in WOC

Direct nursing practice is the core competence of specialist nurses and the primary characteristic of specialist nurses [12,13]. Specialized nurse training in our country starts late and some hospital managers do not attach importance to the use of specialized nurses, so some specialized WOC nurses return to their hospitals after training but are not engaged in specialized nursing work [14]. According to a survey by Zheng et al., only 16.1% of the 87 specialist WOC nurses involved in the investigation were devoted to WOC nursing full-time [15]. In this study, 22.90% of the specialist WOC nurses were engaged in WOC nursing full-time. Compared with the previous investigation, domestic specialist nurses have made great progress. At present, WOC specialist nurses in our country either work full-time or part-time, the majority being the latter. Specialized nursing work does not only involve clinical work but also colostomy positioning, wound debridement, health education, post-operation evaluation of the ostomy, offering related guidance to the patients and their families, etc., all of which require a lot of time and energy; so it is absolutely necessary to institute full-time posts for WOC nurses.

Specialized nursing research involves studying the related problems of the nursing specialty’s own development, including specialized nursing technology, special nursing measures, the nurse-patient relationship, application of new technology, new instruments, and so on. Specialist WOC nursing is developing constantly, and scientific research plays an important role in the development of WOC. Not only can it help solve difficult problems in the workplace and improve their own level of business acumen, but also help to open up new areas of the subject and promote the development of the discipline. In this study, only 24.80% of the specialized WOC nurses obtained scientific research funds, which indicated that the research level of domestic specialist WOC nurses is still weak, and the relevant training needs to be further strengthened.

In this survey, 81.70% of the specialized WOC nurses thought that they had more opportunities for further study after obtaining the specialist WOC nurse qualification. Based on the rapid development of the domestic wound stoma specialty, we should likewise encourage wound stoma specialist nurses to “go out” and learn advanced and mature ideas and techniques from other countries or regions, which could not only promote the exchange between and development of various regions but also make the development of domestic WOC specialty healthy and vigorous [16].

Through this investigation, we found that 58.30% of the specialist nurses were involved in the opening of the specialist WOC outpatient clinics, while 41.70% were not. The reason for this analysis may be the concern that the number of visits to outpatient clinics is too small. Most of these hospitals are specialized hospitals, such as children’s hospitals and psychiatric hospitals. In these kinds of hospitals, there are fewer WOC patients and the WOC specialization is therefore not given enough attention by hospital management. In addition, some specialized WOC nurses have been certified for less than a year, so they worry that they are not skilled and proficient enough, and lack confidence in practice. Therefore, we need to constantly accumulate work experience and update specialized nursing knowledge and skills to deal with complex problems. At the same time, nursing managers should give more encouragement and support in the opening of the specialist WOC outpatient clinics.

Only 26.10% of the specialist WOC nurses in this survey received an additional allowance, and 36.20% have a professional title promotion system. This will inevitably dampen specialist WOC nurses’ enthusiasm toward their work, so we should improve their remuneration when possible, use incentive mechanisms properly, and mobilize the enthusiasm, initiative, and creativity of specialist nurses [17].

## Conclusions

In recent years, the professional development of WOC nurses in China has been rapid, and the number and quality of WOC talent have continuously improved, but there is still a certain gap compared with developed countries, so it is necessary to connect with international standards in the field, learn from advanced training methods used abroad, and further improve WOC nurse training. At the same time, we should encourage specialized nurses to play a more effective role in hospitals after obtaining their qualifications; this not only includes motivation in day-to-day clinical work but also an encouragement to engage in relevant scientific research and to continue learning and updating their specialized knowledge. In addition, the development of WOC specialization cannot be separated from support by the hospital, because these nurses need the full understanding and attention of hospital leaders and doctors. However, it would be much better to further expand the sample size, and actively explore and carry out the corresponding intervention research in the future.
